# ZnO NPs Impair the Viability and Function of Porcine Granulosa Cells Through Autophagy Regulated by ROS Production

**DOI:** 10.3390/antiox13111295

**Published:** 2024-10-25

**Authors:** Yifan Wang, Jing Lv, Guangyu Liu, Qichun Yao, Ziqi Wang, Ning Liu, Yutao He, Dmitry Il, Jakupov Isatay Tusupovich, Zhongliang Jiang

**Affiliations:** 1 Key Laboratory of Animal Genetic, Breeding and Reproduction in Shaanxi Province, College of Animal Science and Technology, Northwest Agriculture and Forestry University, Xianyang 712100, China; 2 Animal Husbandry and Veterinary Station of Zhenba County, Hanzhong 723600, China; 3 Department of Food Security, Agrotechnological Faculty, Kozybayev University, 86, Pushkin Street, Petropavlovsk 150000, Kazakhstan; 4 Department of Veterinary Medicine, Seifullin Kazakh Agro Technical Research University, 62, Zhenis Avenue, Astana 010011, Kazakhstan

**Keywords:** ZnO NPs, ROS, autophagy, granulosa cell, porcine

## Abstract

The zinc oxide nanoparticles (ZnO NPs) is one of the most extensively utilized metal oxide nanoparticles in biomedicine, human food, cosmetics and livestock farming. However, growing evidence suggests that there is a potential risk for humans and animals because of the accumulation of ZnO NPs in cells, which leads to cell death through several different pathways. Nevertheless, the effects of ZnO NPs on porcine granulosa cells (PGCs) and how ZnO NPs regulate the follicular cells are unknown. In this study, we aimed to elucidate the role of ZnO NPs in the porcine ovary by using PGCs. Firstly, we identified the characterization of ZnO NPs used in this study and the results showed that the size of ZnO NPs was 29.0 nm. The results also demonstrated that ZnO NPs impaired cell viability and decreased steroid hormone secretion in PGCs. In addition, ZnO NPs induced reactive oxygen species (ROS) production, leading to oxidative stress of PGCs. Meanwhile, ZnO NPs also triggered autophagy in PGCs by increasing the ratio of LC3-II/LC3-I, along with the expression of SQSTM1 and ATG7. Finally, the results from N-acetylcysteine (NAC) addition suggested that ZnO NPs promoted autophagy through the enhancement of ROS production. In summary, this study demonstrates that ZnO NPs impair the viability and function of PGCs through autophagy, which is regulated by ROS production.

## 1. Introduction

As one of the most prevalently nano metal oxide materials, zinc oxide nanoparticles (ZnO NPs) have been widely used in biomedicine, human food and cosmetics. In animal husbandry, ZnO NPs are utilized as feed additives and an antibiotic substance to facilitate animals’ growth performance [1], increase zinc bioavailability [2] and confer antioxidant benefits [3]. However, growing evidence has raised concerns regarding its potential biosafety risks, particularly on the reproductive system [4], liver [5], neuro cells [6], immune system [7] and cardiac system [8]. The effects of nanoparticles on cell fate largely depend on the morphology and size, as smaller nanoparticles might be more liable to be taken up by tissues and cells [9]. Studies have shown that ZnO NPs added to mouse feeds diminish the sperm count, increase sperm abnormality and lead to the formation of multinucleated giant cells [10]. Additionally, the development of early embryos is inhibited by exposure to ZnO NPs because of oxidative stress, oocyte DNA damage, and the pathological alterations in the ovaries and uterus, inclusive of inflammation, cyst formation, luteinization and endometrial gland hyperplasia [11]. Moreover, the concentrations of estrogen and progesterone in rat serum showed a trend of initial elevation followed by a diminution correlating with increased doses of ZnO NPs in the feedstuffs [12]. Consequently, the detrimental effect of ZnO NPs on animal reproduction has attracted tremendous attention recently.

After sexual maturation, the mammalian ovary contains tens of thousands of follicles. Unfortunately, more than 95% of these follicles undergo atresia at various stages of development [13]. Follicular atresia is a naturally occurring physiological phenomenon prevalent in all stages of follicular development in mammals [14], which are affected by the survival of granulosa cells (GCs), theca cells and/or oocyte, oxidative stress, secretion of steroid hormones etc. Although many studies emphasize the significant role for GCs’ apoptosis in follicle atresia [15,16], a growing range of evidence highlights the importance of GC autophagy during this process. Autophagy constitutes a cellular pathway involved in the degradation of proteins and organelles, which has been confirmed to regulate the differentiation of ovarian granulosa cells by degrading the Wilms’ tumor gene 1 transcription factor, and ovarian function might be impaired by the deficiency in autophagy [17]. Furthermore, research indicates the biological clock’s regulation of autophagy through the suppression of autophagy-related 5 expression mediated by nuclear receptor subfamily 1 group D member 1, thereby holding a crucial role in maintaining GCs’ homeostasis [18].

Studies have shown that the three main factors contributing to the cytotoxicity induced by ZnO NPs are: the release of excessive free zinc ions, the interaction of nanoparticles with the cell surface and the production of excessive reactive oxygen species (ROS) [19]. ROS consist of a family of highly reactive oxygen-containing molecules, including superoxide anions, hydroxyl radicals and hydrogen peroxide, which are involved in regulating signaling pathways that influence cell proliferation and cell death [20]. The relationship between ROS and autophagy is complex and mutually influential; autophagy can be activated in response to oxidative stress induced by excessive ROS production to protect cells from apoptosis. Conversely, impaired autophagy can lead to the accumulation of oxidative stress, further triggering autophagy and creating a vicious cycle. Therefore, ROS can both activate and inhibit autophagic signaling [21]. However, the effects of ZnO NPs on animal reproduction remain poorly understood, and the potential relationship between ROS production in porcine granulosa cells (PGCs) and cellular autophagy has not been comprehensively examined.

In the present study, we hypothesized that ZnO NPs may damage PGCs through autophagy induced by excessive ROS production. To test this hypothesis, we evaluated the status and function of PGCs following ZnO NPs exposure. Additionally, we assessed the levels of autophagy and oxidative stress in PGCs and explored their relationship, aiming to elucidate the interplay between these two processes in PGCs.

## 2. Materials and Methods

### 2.1. Characterization of ZnO NPs

ZnO NPs (#Z820772) were purchased from Shanghai Macklin Biochemical Co., Ltd., Shanghai, China. ZnO NPs were diluted in double-distilled water (ddH_2_O) to 1 mg/mL, followed by vortexing for 1 min and sonication for 30 min to ensure proper dispersion. The size of ZnO NPs was characterized by a transmission electron microscope (TEM, Hitachi, HT7800, Tokyo, Japan). The morphology of ZnO NPs was assessed using a scanning electron microscope (SEM, FEI, Nano SEM-450, Hillsboro, OR, USA). Hydrodynamic sizes and zeta potential analysis were performed using dynamic light scattering (DLS) on the ZEN3600 (Malvern Panalytical, Malvern, UK) to determine the particle size and surface charge of the ZnO NPs.

### 2.2. PGCs Isolation and Culture

PGCs were isolated and cultured as described in our previous work [22]. Porcine ovaries were collected from a local farm abattoir, irrespective of the estrous cycle. For each experiment, at least 20 ovaries were collected and transferred in the laboratory within 1 h in saline containing penicillin (100 U/mL) and streptomycin (100 μg/mL). The ovaries were washed twice with 75% alcohol and then rinsed three times with saline (0.9%, pH < 7, 37 °C). Ovarian follicles measuring 3–5 mm in diameter, with clear follicular fluid, were selected. A 10 mL syringe was used to aspirate the mixture of follicular fluid and granulosa cells. Subsequently, the mixture was centrifuged at 800× *g* for 5 min to separate the granulosa cells. The granulosa cell pellet was resuspended in DMEM/F12 medium (Gibco-BRL, Gaithersburg, MD, USA), and cell number were determined using the trypan blue exclusion method (Solarbio Technology Co., Ltd., Beijing, China). Based on granulosa cell numbers, the cells were then diluted with cell culture medium to a concentration of 5 × 10^5^ cells/mL and were cultured in an incubator at 37 °C with 95% air and 5% CO_2_. The cell culture medium was DMEM/F12 supplemented with 4 ng/mL sodium selenite, 10 mM sodium bicarbonate, 0.1% bovine serum albumin (BSA), 100 U/mL penicillin, 100 μg/mL streptomycin, 1 mM non-essential amino acids, 2.5 μg/mL transferrin, 10 ng/mL insulin and 1 ng/mL FSH (Bioniche Inc., Belleville, ON, Canada). After being cultured for 48 h, 70% culture medium was replaced by fresh medium in each well, after which the treatments were applied.

### 2.3. Cell Viability Assay

After being treated with ZnO NPs and/or N-acetylcysteine (NAC, Beyotime Biological Technology Co., Ltd., Shanghai, China), a CCK-8 assay kit (Beyotime) was used to evaluate the viability of PGCs according to the manufacturer’s instruction. Briefly, 5 × 10^3^ cells/well were seeded into a 96-well plate and incubated for 24 h at 37 °C. Following incubation, CCK-8 solution was added to each well, and the cells were incubated for an additional 3 h until a noticeable colorimetric change occurred. The optical density value at the 450 nm absorbance wavelength was measured by a spectrophotometric microplate reader (Thermo Fisher Scientific, Waltham, MA, USA). Each experiment was performed with at least three independent replicates.

### 2.4. Cell Proliferation Assay

The proliferation of PGCs was evaluated using a Beyo-Click EdU kit (Beyotime). After being treated with ZnO NPs for 24 h, the cell culture medium was substituted with BSA-free DMEM/F12 containing a 1:1000 dilution of EdU, followed by a 4 h incubation at 37 °C. Subsequently, the samples were fixed with 4% paraformaldehyde for 15 min. Following EdU staining, fluorescence microscopy (Olympus Corporation, Tokyo, Japan) was used to capture images of the stained cells. The relative fluorescence intensity was quantitatively analyzed using Image J software (Version 1.51K, National Institutes of Health, USA), with at least five areas selected from each image.

### 2.5. Cell Cycle Assay

The cell cycle of PGCs was assessed using a cell cycle kit (Solarbio, Beijing, China). After 24 h of treatment with ZnO NPs, PGCs were fixed in 75% ethanol at 4 °C overnight. The cells were then washed to remove the ethanol and subsequently incubated with propidium iodide (PI) in the dark environment at 37 °C for 30 min. Cell cycle distribution was analyzed through flow cytometry (BD FACSAria™ III, Franklin Lakes, NJ, USA). The data were processed and analyzed by FlowJo software (Version 10, LLC, Ashland, OR, USA).

### 2.6. Analysis of Steroid Hormone Production

PGCs were seeded into 24-well plates at a concentration of 5 × 10^5^ cells/well. After treatment with ZnO NPs, the cell culture medium was extracted, and the quantification of estradiol (E_2_) and progesterone (P_4_) was measured using an ELISA kit (Ruixin Biological Technology Co., Ltd., Quanzhou, China). Briefly, the medium was centrifuged at 1000× *g* for 10 min at 4 °C and the supernatant was used for steroid hormone assays. To ensure reproducibility, five independent measurements were conducted for each sample. The assay demonstrated coefficients of variations (CVs) within the inter-assay and intra-assay measurements of 4.2% and 8.7% for E_2_, and 3.6% and 5.6% for P_4_, respectively.

### 2.7. Measurement of ROS

ROS in the cells was detected using a DCFH-DA probe (Beyotime) according to the manufacturer’s instructions. Briefly, after 24 h of ZnO NPs treatment in the presence/absence of NAC, the cells were treated with DCFH-DA for 20 min at 37 °C and then washed three times with PBS. Then, the fluorescence intensity of ROS was measured by a fluorescence microscope, capturing at least three random fields of view. For quantitative assessment, flow cytometry was used for quantitative assessment of ROS in the cells based on the guidance. Fluorescence was detected using the FL1 channel with an excitation light of 488 nm and an emission light of 525 nm. The data were subsequently analyzed and interpreted using the FlowJo software.

### 2.8. Measurement of Glutathione Peroxidase Activities and Superoxide Dismutase

The activities of Glutathione Peroxidase (GPX) and Superoxide Dismutase (SOD) in the PGCs were detected by an assay kit (Nanjing Jiancheng Bioengineering Research Institute Co., Ltd., Nanjing, China). Briefly, approximately 5 × 10^5^ cells/well were seeded into 24-well plates. After ZnO NPs treatment for 24 h, the cells were washed twice with PBS and then scraped off using a cell scraper. Subsequently, the cells’ mixture was centrifuged at 1000× *g* for 10 min to remove the supernatant. A BCA (bicinchoninic acid) assay kit (Beyotime) was used to measure the protein concentration according to the manufacturer’s instructions. The activities of GPX and SOD were measured using a spectrophotometer (Thermo) set to a wavelength of 550 nm. The inter-assay and intra-assay CVs were 7.8% and 6.5%, respectively. Each sample was measured at least five times.

### 2.9. Double Staining with MDC and DAPI

Approximately 5 × 10^5^ PGCs per well were seeded in 24-well plates and treated with ZnO NPs for 24 h. Monodansylcadaverine (MDC) was employed as a tracer for autophagic vesicles, with autophagosomes appearing as distinct green dots under a fluorescence microscope. Following treatment, cells were incubated with 0.05 mM MDC (Kaiji Biotechnology Co., Ltd., Nanjing, China) and 1 μg/mL DAPI (4′,6-diamidino-2-phenylindole, Solarbio) at 37 °C for 15 min, followed by immediate fixation with 4% paraformaldehyde for 20 min, according to the manufacturer’s protocol. The stained cells were then observed using a fluorescence microscope. Images were analyzed using ImageJ software (Version 1.51K, National Institutes of Health, USA), with a total of 200 cells per sample assessed. The percentage of cells exhibiting green fluorescence spots was used as an indicator of autophagy levels.

### 2.10. Mitochondrial Membrane Potential Detection

The mitochondrial membrane potential (MMP) of PGCs was detected by a JC-1 kit (Solarbio). Briefly, approximately 5 × 10^5^ cells/well were seeded into 24-well plates and a JC-1 staining working solution was added to each well for 30 min at 37 °C. After being treated with ZnO NPs, the cells were washed three times with PBS. Intercellular fluorescence intensity was measured using a fluorescence microscope, and quantitative analysis of the relative fluorescence intensity was facilitated through the application of ImageJ software. The MMP of PGCs was further confirmed and analyzed by flow cytometry (excitation: 488 nm; emission: 530 nm) within 30 min. Green fluorescence was detected through the FL1 channel, and red fluorescence was detected through the FL2 channel. Image acquisition was randomized and executed across at least three distinct visual fields, and data analysis was conducted using FlowJo software.

### 2.11. RNA Extraction and Quantitative Real-Time PCR

Approximately 1 × 10^6^ PGCs per well were seeded in 6-well plates and treated with ZnO NPs for 24 h. After treatments, the culture medium was removed, and total RNA was extracted from PGCs using TRNzol reagent (Tiangen Biochemical Technology Co., Ltd., Beijing, China), following the manufacturer’s instructions. cDNA was synthesized from the total RNA using HiScript II Q RT SuperMix (+gDNA wiper, Vazyme, Nanjing, China) for Quantitative Real-Time PCR (qRT-PCR) and stored at −20 °C for subsequent experiments. The OD^260^/OD^280^ ratio and the concentration of RNA and cDNA were measured using Nanodrop 2000 (Thermo). qRT-PCR for gene expression was performed using a LineGene 9600 Plus (Bioer Technology Co., Ltd., Hangzhou, China) with ChamQ SYBR qPCR Master Mix (Vazyme) in a 20 μL reaction volume. Transcripts were amplified with the regular thermal cycling parameters (5 min at 95 °C, followed by 40 cycles of 10 s at 95 °C, and 30 s at 60 °C), and melting-curve analyses were conducted to verify product purity. The housekeeping gene β-actin was used to normalize the expression level of mRNA, and the relative gene expression was calculated using the 2^−∆∆Ct^ method. All reactions were performed in at least triplicate to ensure statistical validity. Primer sequences used for qRT-PCR are listed in Table 1.

### 2.12. Western Blot Analysis

Approximately 1 × 10^6^ PGCs per well were seeded in 6-well plates and treated with ZnO NPs for 24 h. After treatment, the culture medium was removed, and the PGCs were washed twice with cold PBS. The cells were then lysed using ice-cold RIPA lysis buffer (Beyotime) containing PMSF (Beyotime) and protease inhibitors (Thermo). Protein concentration was determined using a BCA protein assay kit (Beyotime). A total of 20 µg of protein was separated on 12% SDS-PAGE gels and transferred to polyvinylidene difluoride (PVDF) membranes (Millipore, Billerica, MA, USA) using a Bio-Rad wet blot transfer cell apparatus with transfer buffer (39 mM glycine, 48 mM Tris-base, 1% SDS, 20% methanol, pH 8.3). The PVDF membranes were then blocked with QuickBlock™ Western’s blocking buffer (Beyotime) for 1 h at room temperature, followed by overnight incubation at 4 °C with primary antibodies, including β-Actin (Mouse polyclonal to ACTB, 1:5000, Proteintech Group, Inc., Chicago, IL, USA), LC3 (Rabbit polyclonal to LC3, 1:2000, Abcam, Cambridge, UK), SQSTM1 (Rabbit polyclonal to SQSTM1 1:2000, Abcam), BECN (Rabbit polyclonal to BECN1, 1:2000, Abcam), ATG7 (Rabbit polyclonal to ATG7, 1:10,000, Abcam), STAR (Rabbit polyclonal to STAR, 1:1000, Proteintech Group, Inc., Chicago, IL, USA), GPX1 (Rabbit polyclonal to GPX1, 1:5000, Proteintech), CYP11A1 (Rabbit polyclonal to CYP11A1, 1:1000, ABclonal Biotech Co., Ltd., Wuhan, China) and SOD2 (Rabbit polyclonal to SOD2, 1:1500, Wanleibio Co., Ltd., Shenyang, China). After three washes in TBST (150 mM NaCl, 2 mM KCl, 25 mM Tris, 0.05% Tween20, pH 7.4), the membranes were incubated for 1 h at room temperature with HRP-conjugated anti-rabbit IgG (1:4000, Sungene Biotechnology, Tianjin, China) or anti-mouse HRP-conjugated IgG (1:4000, Sungene), diluted in QuickBlock™ Secondary Antibody Dilution Buffer (Beyotime). Following three additional washes in TBST, protein bands were visualized using enhanced chemiluminescence (ECL, Millipore) with the ChemiDoc XRS+ imaging system (Bio-Rad Laboratories, Inc., Hercules, CA, USA). Semi-quantitative analysis was performed using ImageJ software (Version 1.51K, National Institutes of Health, USA) and Image Lab Software (Version 3.0, Bio-Rad, Hercules, CA, USA). Representative images were selected from at least three independent experiments, and relative protein expression levels were normalized to β-actin for comparison across different proteins.

### 2.13. Statistical Analysis

All experiments were repeated at least three times, with each experiment performed in triplicate using at least five samples per treatment to ensure repeatability in this study. Independent *t*-tests were used to evaluate the significance of differences between groups. One-way ANOVA was used to test the main effects among treatments, with the culture replicate included as a random variable in the F-test. The Tukey–Kramer HSD test was used to analyze differences between group means (GraphPad Prism version 9.0, GraphPad Software Inc., San Diego, CA, USA). If the data were not normally distributed (as determined by the Shapiro–Wilk test), they were log-transformed. Data are presented as the means ± S.D. Statistical significance was set at *p* < 0.05.

## 3. Results

### 3.1. Characteristics of ZnO NPs

**Figure 1:**
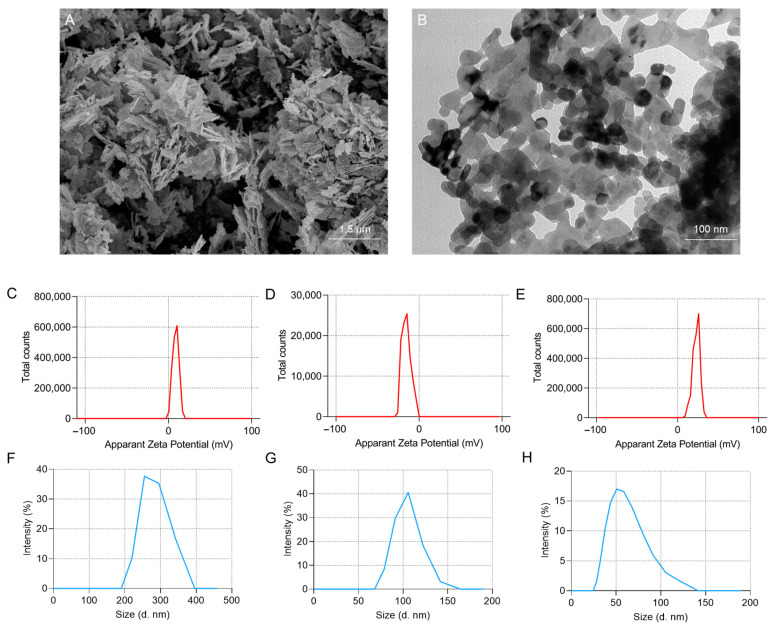
Identification and characterization of ZnO NPs: (**A**) Image of ZnO NPs captured by SEM. (**B**) Image of ZnO NPs captured by TEM. (**C**–**H**) DLS analysis showing the zeta potential of ZnO NPs dispersed in PBS (**C**), 0.9% NaCl (**D**) and ddH_2_O (**E**), and the hydrodynamic size of ZnO NPs dispersed in PBS (**F**), 0.9% NaCl (**G**) and ddH_2_O (**H**), respectively.

To ensure the usability of the ZnO NPs, their characteristics were first assessed. SEM imaging revealed that ZnO NPs were snowflake-shaped and evenly distributed (Figure 1A), while TEM analysis showed that the average size of ZnO NPs was 29.0 ± 3.5 nm (Figure 1B). In addition, the results from DLS indicated that the zeta potential values of ZnO NPs in liquids of PBS, 0.9% NaCl and ddH_2_O were 8.73 ± 3.64 mV, −15.29 ± 5.26 mV and 22.96 ± 4.45 mV, respectively (Figure 1C–E), and the hydrodynamic sizes were 280.26 ± 21.60 nm, 103.38 ± 14.48 nm and 58.76 ± 19.59 nm, respectively (Figure 1F–H). These findings suggest that ZnO NPs exhibit better stability in ddH_2_O; thus, the mixture of ZnO NPs dispersed in ddH_2_O was used for subsequent experiments.

### 3.2. ZnO NPs Impair the Viability and Proliferation of PGCs

**Figure 2:**
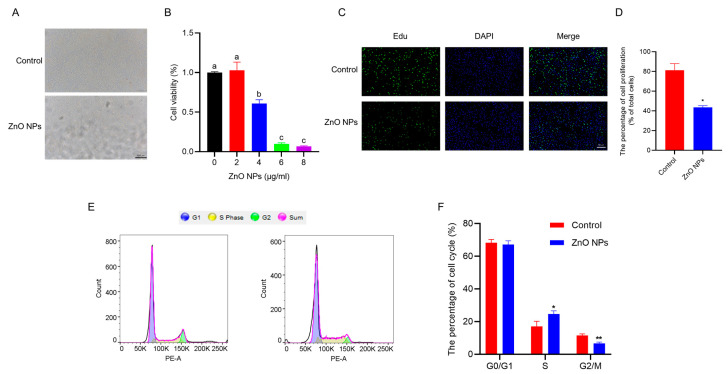
Effects of ZnO NPs on PGCs: (**A**) Images of PGCs captured under a regular light microscope after 24 h of ZnO NPs treatment. (**B**) Cell viability measured by CCK8 assay following 24 h of ZnO NPs treatment. (**C**,**D**) Proliferation of PGCs detected by EdU staining, with statistical analysis. (**E**) Cell cycle distribution of PGCs measured by flow cytometry. (**F**) Percentage of cells in different phases of the cell cycle. Different lowercase letters indicate significant difference among groups (*p* < 0.05). * indicates *p* < 0.05 and ** indicates *p* < 0.01.

To investigate the effects of ZnO NPs on PGCs in vitro, the viability of PGCs treated with ZnO NPs was initially assessed. The results indicated a dose-dependent decrease in PGC viability following treatment with ZnO NPs at concentrations of 0, 2, 4, 6 and 8 µg/mL (Figure 2B). A significant reduction in cell number was observed at a concentration of 4 µg/mL of ZnO NPs (Figure 2A). Based on these results, a concentration of 4 μg/mL of ZnO NPs was selected for further experiments. Additionally, proliferation assays were conducted to evaluate the effect of ZnO NPs exposure on PGCs, revealing a significant suppression in the proliferation of PGCs (Figure 2C,D). To further understand the mechanism of ZnO NPs’ effect on cellular proliferation, the cell cycle distribution of PGCs on the control group and ZnO NPs treatment (Figure 2F) were analyzed. The results showed that ZnO NP exposure led to a notable increase in the proportion of cells in the S phase and a decrease in the proportion of cells in the G2/M phase (Figure 2F). These findings suggest that ZnO NPs impair the viability and proliferation of PGCs.

### 3.3. ZnO NPs Suppress Steroid Hormone Secretion in PGCs

**Figure 3:**
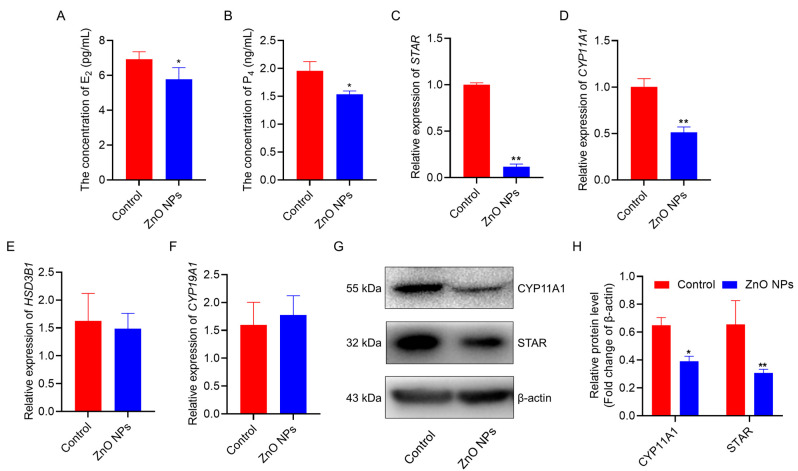
ZnO NPs suppress steroid hormone secretion in PGCs: (**A**,**B**) Concentrations of E_2_ (**A**) and P_4_ (**B**) in PGCs following treatment with ZnO NPs. (**C**–**F**) Relative gene expression of *STAR* (**C**), *CYA11A1* (**D**), *HSD3B1* (**E**) and *CYP19A1* (**F**) in PGCs. (**G**,**H**) Western blot analysis of protein levels in PGCs, with statistical results for CYP11A1 and STAR protein levels. * indicates *p* < 0.05 and ** indicates *p* < 0.01.

One of the critical functions of ovarian granulosa cells is to synthesize and secrete steroid hormones, which are essential for follicle maturation. To investigate the effects of ZnO NPs on the steroidogenic synthesis in PGCs, we assessed the concentrations of E_2_ and P_4_ in the culture medium. Our results showed that the E_2_ and P_4_ concentrations in the culture medium of PGCs exhibited a significant reduction (Figure 3A,B). Additionally, we quantified the mRNA expression levels of genes associated with steroid hormone biosynthesis, revealing a repression in the mRNA expression levels of *steroidogenic acute regulatory protein* (*STAR)* and *cytochrome P450 family 11 subfamily A member 1* (*CYP11A1)* (Figure 3C,D), with no significant changes in the expression of *hydroxy-delta-5-steroid dehydrogenase, 3 beta- and steroid delta-isomerase 1* (*HSD3B1)* and *cytochrome P450 family 19 subfamily A member 1* (*CYP19A1)* (Figure 3E,F). Furthermore, Western blot analysis confirmed that ZnO NPs inhibited the expression of CYP11A1 and STAR proteins (Figure 3G,H). Together, these observations indicate ZnO NPs suppress steroid hormone secretion in PGCs.

### 3.4. ZnO NPs Induce Oxidative Stress to Damage Mitochondrial Membrane in PGCs

**Figure 4:**
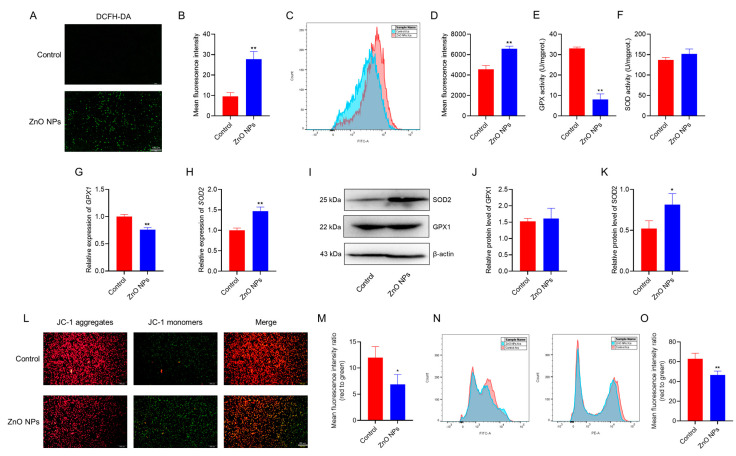
ZnO NPs induce oxidative stress, leading to mitochondrial membrane damage in PGCs: (**A**) Fluorescence images of ROS in PGCs stained with DCFH-DA. (**B**) Statistics results of ROS fluorescence intensity. (**C**) Intracellular ROS production in PGCs assessed by flow cytometry. (**D**) Statistics results of intracellular ROS in PGCs. (**E**,**F**) Activity of GPX (**E**) and SOD (**F**) in PGCs. (**G**,**H**) Relative expression of *GPX1* (**G**) and *SOD2* (**H**) genes in PGCs. (**I**–**K**) Protein level of GPX1 and SOD2 measured by western blotting (**I**), with statistical results for GPX1 (**J**) and SOD2 (**K**) protein level in PGCs. (**L**) Images of MMP in PGCs obtained from JC-1 staining. (**M**) Ratio of red to green fluorescence intensity from JC-1 staining. (**N**) MMP in PGCs assessed by flow cytometry. (**O**) Statistics results of relative fluorescence intensity in PGCs. * indicates *p* < 0.05 and ** indicates *p* < 0.01.

Oxidative stress is one of the most important factors affecting cell viability and function. In order to understand the roles of ZnO NPs in PGCs, we measured intracellular ROS levels and assessed their impact on mitochondrial activity. As shown in Figure 4A, florescence images of PGCs stained with DCFH-DA demonstrated that ROS production significantly increased in cells exposed to ZnO NPs (Figure 4B). Flow cytometry results further indicated that ZnO NPs induced oxidative damage in PGCs (Figure 4C,D). In addition, the activity of GPX in PGCs was reduced (Figure 4E), while SOD activity remained unaffected (Figure 4F). At the gene expression level, *glutathione peroxidase 1* (*GPX1)* expression was downregulated (Figure 4G), whereas *superoxide dismutase 2* (*SOD2)* expression was upregulated (Figure 4H) in PGCs treated with ZnO NPs. The proteins of GPX1 and SOD2 were measured, and the results showed that the exposure to ZnO NPs of PGCs did not alter GPX1 protein expression, while it increased SOD2 protein expression (Figure 4I–K). Mitochondria are the primary sources of intracellular ROS, so to further explore the mechanism of ROS production in PGCs exposed to ZnO NPs, we assessed the MMP following ZnO NPs treatment. The results of fluorescence staining indicated that ZnO NPs decreased the MMP in PGCs (Figure 4L,M), and this reduction was further confirmed by flow cytometry (Figure 4N,O). Together, these findings indicate that ZnO NPs induce increased ROS production, leading to oxidative stress and mitochondrial damage in PGCs.

### 3.5. ZnO NPs Promote Autophagy in PGCs

**Figure 5:**
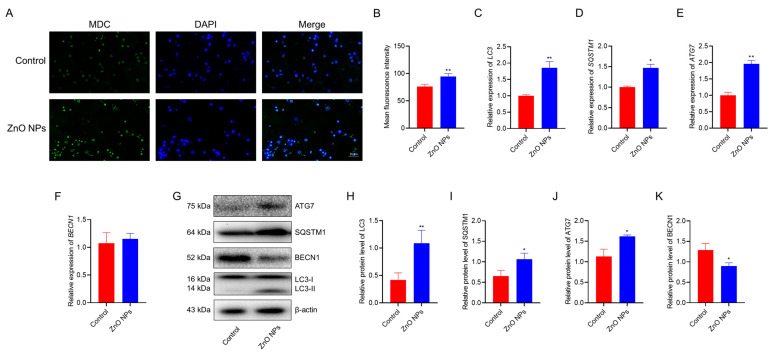
ZnO NPs promote autophagy in PGCs: (**A**) Image of PGCs stained with MDC. (**B**) Statistics results of MDC fluorescence intensity. (**C**–**F**) Relative expression of autophagy-related genes: *LC3* (**C**), *SQSTM1* (**D**), *ATG7* (**E**) and *BECN1* (**F**). (**G**) Western blotting analysis of autophagy marker proteins. (**H**–**K**) Statistics results for LCB (**H**), SQSTM1 (**I**), ATG7 (**J**) and BECN1 (**K**) protein levels. * indicates *p* < 0.05 and ** indicates *p* < 0.01.

The damage to the mitochondrial membrane may trigger cellular autophagy. In this study, we speculated that autophagy might occur in PGCs treated with ZnO NPs, based on the previous results. To verify this speculation, autophagy in PGCs was detected using MDC staining, and the results showed that ZnO NPs treatment significantly induced autophagy (Figure 5A,B). Furthermore, mRNA expression of autophagic markers was measured in PGCs, and the data obtained from qRT-PCR indicated a significant upregulation in the gene expression of *microtubule associated protein 1 light chain 3* (*LC3)*, *sequestosome 1* (*SQSTM1)* and *autophagy-related 7* (*ATG7)* following ZnO NP treatment, while beclin 1 (*BECN1)* mRNA levels were unaffected by ZnO NPs (Figure 5C–F). Additionally, the levels of LC3, SQSTM1, ATG7 and BECN1 proteins were assessed (Figure 5G). The results revealed that ZnO NPs treatment notably increased the ratio of LC3-II/LC3-I (Figure 5H) and the expression of SQSTM1 (Figure 5I) and ATG7 (Figure 5J), while decreasing the expression of BECN1 protein in PGCs (Figure 5K). Collectively, these findings clearly indicate that ZnO NPs promote autophagy in PGCs.

### 3.6. NAC Alleviated the Autophagy Induced by ZnO NPs in PGCs

**Figure 6:**
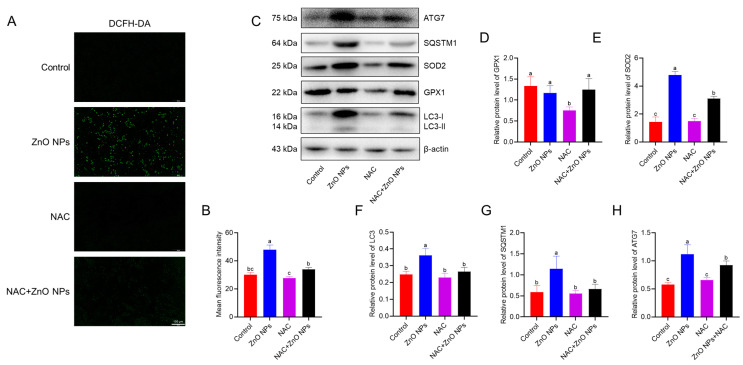
The effects of NAC on PGCs’ autophagy treated by ZnO NPs: (**A**) Fluorescence images of ROS in PGCs treated with NAC, stained with DCFH-DA. (**B**) Statistics results of ROS fluorescence intensity. (**C**) Western blotting analysis of antioxidant protein and autophagy marker protein expression. (**D**–**H**) Statistics results of GPX1 (**D**), SOD2 (**E**), LC3B (**F**), SQSTM1 (**G**) and ATG7 (**H**) protein levels. Different lowercase letters indicate significant difference among groups (*p* < 0.05).

To investigate whether autophagy in PGCs is induced by ROS, the antioxidant NAC was used to reduce ROS levels, and autophagy-related markers were subsequently assessed in this study. First, ROS levels in PGCs were determined using DCFH-DA staining. The results indicated that the intracellular ROS levels were significantly decreased in PGCs treated with ZnO NPs due to the presence of NAC (Figure 6A,B). Next, the expression levels of antioxidant enzymes and autophagy-related proteins were evaluated (Figure 6C). As shown in Figure 6D, the expression of GPX1 was suppressed by NAC, while the combination of ZnO NPs and NAC did not alter GPX1 protein levels in PGCs. With comparison to the treatment of ZnO NPs, NAC dramatically reduced the expression of SOD2, even when ZnO NPs and NAC were used together (Figure 6E). Regarding autophagy-related proteins, both NAC alone and in combination with ZnO NPs reduced the levels of LC3, SQSTM1 and ATG7 proteins (Figure 6F–H). Collectively, these results suggest that NAC effectively inhibited the autophagy induced by ZnO NPs in PGCs through the reduction of intracellular ROS levels.

## 4. Discussion

With the widespread use of ZnO NPs in biomedicine, food, cosmetics and animal husbandry as an additive and antibiotic substance, the potential impact of ZnO NPs on human health and environmental safety has attracted significant attention from consumers and researchers [23]. In the animal reproductive system, recent studies have shown that ZnO NPs can disrupt meiosis in mouse oocytes by inhibiting glucose-regulated protein 78 and inducing endoplasmic reticulum stress [24]. Additionally, Qian et al. suggest that ZnO NPs may adversely affect the male reproductive system by damaging the blood–testis barrier [25]. Our previous study indicated that ZnO NPs cause mitochondrial damage in spermatocytes and reveled the mechanism of ferroptosis induced by ZnO NPs through increased intracellular chelatable iron content and lipid peroxidation levels [26]. In this study, we provide direct evidence that ZnO NPs promote autophagy in PGCs. Specifically, we demonstrate that (1) ZnO NPs induced oxidative stress and reduced MMP in PGCs, and (2) intracellular ROS triggered autophagy in PGCs, leading to inhibited proliferation and hormone secretion. Collectively, these results indicate that ZnO NPs impair the viability and function of PGCs through cellular autophagy regulated by ROS.

The biological characteristics, safety and cellular interactions of nanoparticles are influenced by their physicochemical properties, including particle size, shape, surface coating and absorptivity. It is well documented that the intrinsic characteristics of nanoparticles are positively correlated with their adverse effects [27], with a smaller particle size and larger surface area leading to higher reactivity potential and toxicity in cells [28]. Additionally, Xiao et al. demonstrate that natural organic matter significantly impacts the bioaccumulation kinetics and tissue distribution of silver nanoparticles (AgNPs) in aquatic organisms, reducing their bioaccumulation and absorptivity in zebrafish and thereby mitigating AgNPs toxicity [29]. Under similar conditions, ZnO NPs are more widely distributed across various tissues and show higher absorption rates compared to other nanoparticles [30]. Therefore, we first characterized ZnO NPs that we used by using TEM and SEM. Our results indicated that the ZnO NPs used in this study have smaller average sizes and a lower surface area, which might lead to higher mobility and reactivity potential.

In ovaries, follicles are recruited and develop to preovulatory follicles in response to gonadotropin stimulation. Ovarian granulosa cells, as one of the main cell types in the follicle, are responsible for synthesizing and secreting steroid hormones, which play a crucial role in promoting follicular growth and oocyte maturation [31], and the proliferation of GCs themselves [32]. In the current study, based on the characterization of the nanoparticles used, upon treatment of PGCs with ZnO NPs at a concentration of 4 μg/mL for 24 h, cell viability decreased to 60.99%. In addition, our results indicated that ZnO NPs significantly inhibited the proliferation of PGCs through the disruption of the cell cycle. Specifically, the proportion of cells in the G0/G1 phase remained unchanged, while the proportion of cells in the S phase increased and the proportion in the G2/M phase decreased. However, the exact mechanism by which ZnO NPs affect the proliferation and cell cycle of PGCs requires further investigation.

Until the secondary follicle stage, GCs respond to follicle-stimulating hormone and upregulate CYP19A1 expression to synthesize E_2_ and/or P_4_ [33]. Both E_2_ and P_4_ play crucial roles during female animal estrus, follicular growth and rapture, and the maintenance of pregnancy [34]. However, it has been shown that nanoparticles regulate the synthesis and secretion of E_2_ and P_4_ in different animal models. In mice, Ramses et al. found that exposure to 5 µg/mL of ZnO NPs increased E_2_ levels by inducing *CYP19A1* mRNA expression through the release of free Zn ions [35]. In contrast, in fish, high concentrations of AgNPs combined with 17α-ethinylestradiol caused a decrease in estrone levels in female fish and androstenedione levels in plasma while also triggering synergistic effects on plasma steroid hormone concentrations in juvenile turbots [36]. STAR plays a key role during steroid hormone synthesis by transporting cholesterol from the outer to the inner mitochondrial membrane in steroidogenic cells, a fundamental rate-limiting step in steroid hormone production [37]. CYP11A1 is a gene encoding the cholesterol side-chain cleavage enzyme, which primarily catalyzes the conversion of cholesterol to pregnenolone, marking the initial phase of steroid hormone synthesis [38]. Our current results demonstrated that ZnO NPs suppressed both E_2_ and P_4_ concentrations in PGCs in vitro, which contrasts with the findings of Ramses et al. in mice. Additionally, ZnO NPs suppressed the expression of *STAR* and *CYP11A1* mRNA in PGCs, as well as their corresponding protein levels.

Mitochondria play a crucial role in various cellular activities, including growth, proliferation, differentiation and death. However, mitochondria are also the main target for the toxic effects of many nanoparticles [39]. Many nanoparticles initially cause cell damage at a sublethal stage, during which cells can either recover or cross a “point of no return” [40]. This critical point is associated with a key separation between the outer and inner mitochondrial membranes, followed by the irreversible loss of oxidative phosphorylation capacity [41]. Similar to the effects of many nanoparticles, such as polystyrene nanoparticles [42] and AgNPs [43] on GCs, ZnO NPs also led to a decrease in MMP in PGCs in this study. This mitochondrial damage resulted in the release of excessive ROS, which further exacerbated mitochondrial damage within the cells. In agreement with other studies [44,45,46] and our previous research [26], the current results showed that ZnO NPs increased the production of ROS in PGCs, which disrupted the balance between free radical production and antioxidant system activity. This imbalance causes various types of cellular damage, including a decrease in MMP in PGCs.

Recent research has documented that the loss of MMP can damage various antioxidant enzymes within cells due to nanoparticle-induced toxicity [47]. These antioxidant enzymes are crucial for eliminating excessive ROS and maintaining redox homeostasis in cells. SOD is the first enzyme to address oxyradicals, catalyzing the dismutation of the highly toxic superoxide radical into oxygen and hydrogen peroxide. Hydrogen peroxide is further metabolized into water by either Catalase or GPX using glutathione and is maintained by the GPX reaction. Studies have shown that under conditions of high SOD activity and low GPX activity, hydrogen peroxide can accumulate in mitochondria, leading to oxidative stress or accelerating the transformation of cells into tumor cells [48]. ROS also play a regulatory role in the physiological arrest during oocyte ovulation, with moderate increases in ROS levels capable of resuming meiosis in arrested oocytes [49]. Several studies have demonstrated that physiological levels of ROS are beneficial for folliculogenesis, maintaining the function of GCs within follicles, oocyte maturation and embryonic development [50]. Despite the follicle’s robust antioxidant capacity prior to ovulation, excessive ROS can overwhelm this defense mechanism, leading to oxidative stress within the follicular cells [51]. In the present study, exposure to ZnO NPs significantly reduced intracellular GPX activity, while SOD activity exhibited an increasing trend without a significant change. The relative expression of the antioxidant enzyme gene *GPX1* was significantly downregulated, whereas the expression of *SOD2* was significantly upregulated. Western blot analysis further confirmed a significant increase in SOD2 protein levels within the cells. In the present study, the treatment of ZnO NPs induced the production of ROS, disrupting mitochondrial homeostasis and leading to the loss of MMP in PGCs. Combined with the dysregulation of the antioxidant enzyme network, this suggests that the cells are entering a state of oxidative stress, which is a primary factor contributing to the proliferation arrest of PGCs caused by ZnO NPs.

Autophagy is one of the cellular processes involved in maintaining cellular homeostasis, differentiation and survival. Both cell survival and death can be related to autophagy when cells are subjected to stressful conditions [52]. The influence of NPs on autophagy can be categorized into two distinct outcomes: an increase in autophagosome formation and flux, or autophagic dysfunction [41]. In both scenarios, NPs typically increase the LC3 protein level, which aligns with our results. However, in the case of autophagic dysfunction, there is also an increase in SQSTM1, a protein involved in cargo delivery to the autophagosome by binding ubiquitinated proteins and LC3—that is no longer degraded via autophagy [53]. Here, we demonstrated that ZnO NPs activated PGCs’ autophagy by upregulating the ratio of LC3-II/LC3-I and increasing the expression of SQSTM1 and ATG7 at both the mRNA and protein levels.

Many studies have shown that autophagy is influenced by various factors and environmental stimuli, including oxidative stress, starvation and epigenetic regulation [54]. Recent studies have confirmed that the formation of autophagosomes and autophagic degradation is related to the production of ROS; conversely, autophagy may also reduce ROS concentration and mitigate oxidative stress by eliminating protein aggregates and damaged organelles [55,56]. In this study, the role of ROS in the autophagy of PGCs was investigated. Our results showed that NAC addition decreased the production of excess ROS and reversed the expression patterns of antioxidant enzymes GPX1 and SOD2, as well as autophagy-related proteins LC3, SQSTM1 and ATG7. Together, these findings indicate that the ZnO NPs-induced autophagy of PGCs can be altered by reducing excess ROS production and restoring oxidative balance.

## 5. Conclusions

In conclusion, we demonstrated for the first time that ZnO NPs promoted autophagy by increasing ROS production, which impaired the viability and function of PGCs. These findings provide important insights into the effects of ZnO NPs and their potential applications in animal husbandry.

## Figures and Tables

**Table 1:** Primers used for Quantitative Real-Time PCR.

Gene	Primer Sequence (5′-3′)	Genebank No.	Size (bp)
*β-actin*	F: TGCGGGACATCAAGGAGAAG	XM_003124280.5	216
	R: AGTTGAAGGTGGTCTCGTGG		
*SQSTM1*	F: AAGCTGAGACATGGGCACTT	XM_003123639.4	173
	R: ACACTCTCCCCTACGTTCTTG		
*ATG7*	F: AGATTGCCTGGTGGGTGGT	NM_001190285.1	140
	R: GGGTGATGCTGGAGGAGTTG		
*LC3*	F: GCCTCTCAGGAGACTTTCGG	NM_001190290.1	214
	R: GAGCTCCGTTTTTCTGCGTG		
*BECN1*	F: AGGAGCTGCCGTTGTACTGT	NM_001044530.1	189
	R: CACTGCCTCCTGTGTCTTCA		
*S* *T* *AR*	F: CGTCGGAGCTCTCTTCTTGG	NM_213755.2	124
	R: CCTCCTGGTTGCTGAGGATG		
*CYP11A1*	F: CGAAGGACCCAACCCAGAACGA	NM_214427.1	237
	R: CCAGAACCCTGCTGCTTGATGC		
*HSD3B1*	F: ATTTCTCGGTGCCCAGGTTT	NM_001004049.2	180
	R: GCTCTGGAGCTTAGAAAATTCCTC		
*CYP19A1*	F: AGAAGGGTCACAACAAGACAG	NM_214429.1	125
	R: AGGCACAACTTCAGACACCAT		
*SOD2*	F: GGCCTACGTGAACAACCTGA	NM_214127.2	126
	R: TGATTGATGTGGCCTCCACC		
*GPX1*	F: CTAGCAGTGCCTAGAGTGCC	NM_214201.1	142
	R: CGCCCATCTCAGGGGATTTT		

## Data Availability

The data are contained within this article.
